# Functions and Sensors of Smart Walkers From 2015 to 2024: Scoping Review

**DOI:** 10.2196/78480

**Published:** 2026-05-26

**Authors:** Nicole Strutz, Hanna Brodowski, Stephan Schulze

**Affiliations:** 1 MUC.HEALTH, Munich University of Applied Sciences, Landsbergerstraße 187, Munich, Bavaria, 80687, Germany, 49 89 1265-236; 2 Department of Orthopedic and Trauma Surgery, Martin Luther University Halle-Wittenberg, Halle (Saale), Germany; 3 Department of Physiotherapy, Pain and Exercise Research Luebeck (P.E.R.L.), Institute of Health Sciences, Universität zu Lübeck, Lübeck, Germany

**Keywords:** walking aid, navigation, gait analysis, older adults, clinical setting, user feedback, observer feedback

## Abstract

**Background:**

Early mobilization and mobility are essential components of the recovery process following surgery and trauma-related hospitalization. In addition to personalized support from physiotherapists and health care professionals, assistive devices such as walkers play a crucial role in facilitating safe and effective mobility.

**Objective:**

This scoping review aims to provide a comprehensive overview of the current state of the literature on the design, sensor technologies, and functional applications of smart walkers and to assess the extent to which existing studies reflect clinical use cases.

**Methods:**

Peer-reviewed English articles published between 2015 and 2024 were identified by searching PubMed, CINAHL, SSCI, and IEEE, focusing on the topic of smart walkers. Secondary analyses and walkers with 2 wheels or fewer were excluded in abstract screening. Study screening and selection were performed according to the Joanna Briggs Institute guidelines for scoping research and reported following the PRISMA-ScR (Preferred Reporting Items for Systematic Reviews and Meta-Analyses extension for Scoping Reviews) guidelines. The Rayyan systematic review management software was used for study selection. The articles included were analyzed with respect to the sensor technologies used, their functional capabilities, and their application scenarios.

**Results:**

Of the 800 articles screened, 44 (5.5%) met the inclusion criteria. Most of these articles were research reports (n=36, 81.8%) and were conducted in laboratory-based environments (n=30, 68.2%). Most studies evaluated smart walkers in asymptomatic populations (n=29, 65.9%), with half (n=22, 50%) involving younger adults. Among the sensor modalities reported, camera-based and light detection and ranging–based sensors were most prevalent for half of the implementations. Light detection and ranging–based sensors can be categorized according to their primary functions: gait analysis (n=11, 25%), collision detection (n=9, 36%), and navigation (n=5, 11.4%). Load sensors (n=10, 22.7%) and ultrasonic sensors (n=11, 25%) were among the most frequently cited sensor modalities in the literature. Load sensors, also known as force sensors, are integrated into the handlebars, frame, forearm supports, or chest pads of smart walkers. These sensors measure the user’s load, providing essential data for calculating body weight support or inferring the user’s intention to move.

**Conclusions:**

The smart walkers described in the literature were predominantly tested in asymptomatic and younger populations. Bridging the gap between current laboratory-based research and real-world clinical environments, as well as the daily lives of end users, remains a critical objective. Addressing the specific needs of older adults through comprehensive requirements analyses and iterative testing continues to be an ongoing challenge, yet these processes can serve as integral components of research and development projects.

## Introduction

In contemporary society, we are confronted with the dual challenges of an aging population and evolving demands in daily routines. These challenges are compounded by age-related impairments in walking ability, often due to lower limb discomfort [1] and/or compromised balance [2], which necessitate the use of mobility aids. The selection of appropriate mobility aids (eg, wheelchair, crutches, and walker) has to be tailored to the individual’s remaining functional capacities [3].

Such aids are essential not only for addressing mobility issues but also for mitigating the long-term consequences of reduced compensatory ability (eg, muscle atrophy, altered gait patterns, and further decline in balance) [4]. Without appropriate intervention, this progressive decline may lead to a further reduction in residual capacities, potentially creating a cycle of dependency and physical deterioration [5].

Mobility aids are not restricted to indoor use; they are also vital for maintaining autonomy and mobility outside the home, such as for visiting medical practitioners, shopping, or engaging in social activities. The primary target group for these devices is older adults, for whom mobility aids play a vital role in maintaining participation in daily life and preserving independence in later years [6].

In hospital settings, individuals remain dependent on mobility aids to prevent the adverse effects of immobility, including muscle atrophy, joint contractures, and psychological deterioration. Although clinical settings typically prioritize disease diagnosis and treatment, preserving mobility and promoting early mobilization [7,8] with the aid of assistive devices is an essential objective for health care providers [9]. In this context, walkers and other gait-assisting devices play a crucial role, especially in early mobilization.

Technological advancements in mobility aids have been ongoing for several years, leading to the development of functionally enhanced walkers. These devices now integrate various assistive technologies, such as motorized support for propulsion, navigation systems, balance detection, counter-steering mechanisms in response to balance loss, and compensatory features for sensory deficits [10,11]. Additionally, smart walkers may serve as valuable tools in biomechanical diagnostics within orthopedic clinics when gait and/or postural analysis are implemented or for individualized risk prediction of falls [12]. According to Valadão et al [13], smart walkers are “...walkers that contain, besides the mechanical structure to support the user, electronics, control systems and sensors, in order to allow a better user experience and minimize the risks of falling.”

The aim of this scoping review is to identify and map the current applications of functionally enhanced walkers, commonly referred to as smart walkers. The study aims to answer the following primary research question: What are the current functions of smart walkers, and in which settings are they applied?

Secondary research questions include the following: (1) Which types of sensors are integrated into smart walkers? (2) What are the specific application scenarios for these devices, including contextual settings and target user groups?

## Methods

### Study Design

This scoping review was conducted in accordance with the Joanna Briggs Institute guidelines for scoping research and reported following the PRISMA-ScR (Preferred Reporting Items for Systematic Reviews and Meta-Analyses extension for Scoping Reviews) [14] guidelines. The literature search was performed in January and February 2025. The study protocol was registered on the Open Science Framework platform (registration number: 10.17605/OSF.IO/CTPF4).

### Information Sources and Search Strategy

The search encompassed publications from January 2015 to December 2024, using a predefined search string. The search string for the PubMed database is provided in Textbox 1. The search strings for 4 other databases can be found in Multimedia Appendix 1. An initial search was performed in PubMed, SSCI, CINAHL, IEEE, and Cochrane Library, which informed the development of a comprehensive search strategy subsequently applied across all sources. Final searches were conducted in the 5 databases. After removing duplicates and screening titles and abstracts, full-text reviews were conducted.

Textbox 1. **Search string for the PubMed database.**

**PubMed**

1. Walker* [Title/Abstract]
2. rollator* [Title/Abstract]
3. device supported [Title/Abstract]
4. walker* [MeSH Terms]
5. #1 OR #2 OR #3 OR #4
6. smart [Title/Abstract]
7. intelligent [Title/Abstract]
8. robot* [Title/Abstract]
9. artificial [Title/Abstract]
10. ai [Title/Abstract]
11. #6 OR #7 OR #8 OR #9 OR #10
12. DNA [Title/Abstract]
13. device-based [Title/Abstract]
14. molecular [Title/Abstract]
15. Covid* [Title/Abstract]
16. exoskeleton
17. teaching
18. walker* and avant*
19. #12 OR #13 OR #14 OR #15 OR #16 OR #17 OR #18
20. #5 AND #11 NOT #19

### Inclusion and Exclusion Criteria

Articles published in English in peer-reviewed journals that focused on smart walkers and independent walking users were included. Exclusion criteria encompassed secondary analyses (eg, reviews), smart walking aids with 2 wheels or fewer, or studies where the term “walker” referred to nonsmart devices.

### Study Selection

The Rayyan (Rayyan Systems Inc) systematic review management software was used for study selection. Four independent reviewers (HB, MW, SS, and NS) initially screened titles and abstracts to exclude articles that did not meet the inclusion criteria. Subsequently, full-text screening was performed independently by 2 of the aforementioned reviewers. Discrepancies were resolved through discussion with a third reviewer until consensus was achieved.

### Data Extraction

Data from each included study were extracted independently by HB, MW, NS, and SS using a structured data extraction framework. Any conflicts were resolved through discussion with the principal investigator (NS). The reviewers’ professional backgrounds include doctoral (PhD) degrees in physiotherapy and health sciences, with experience in health technologies related to mobility in older patients, as well as physiotherapy care and therapy for older patients. They also have research experience and have conducted studies on health technologies that address mobility. Furthermore, the researchers are sports scientists and nursing professionals with expertise in geriatric care for older clients and 10 years of experience in clinical gait analysis.

Data extracted from each publication included general publication details (year, author, journal, and country), study population and setting, and characteristics of smart walkers (hardware, software, and technological features such as sensors and feedback mechanisms), as well as technological features (sensors and feedback mechanisms). These data are presented in the *Results* section.

As per the scoping review methodology, the quality of included studies was not appraised.

## Results

### Overview

**Figure 1.**
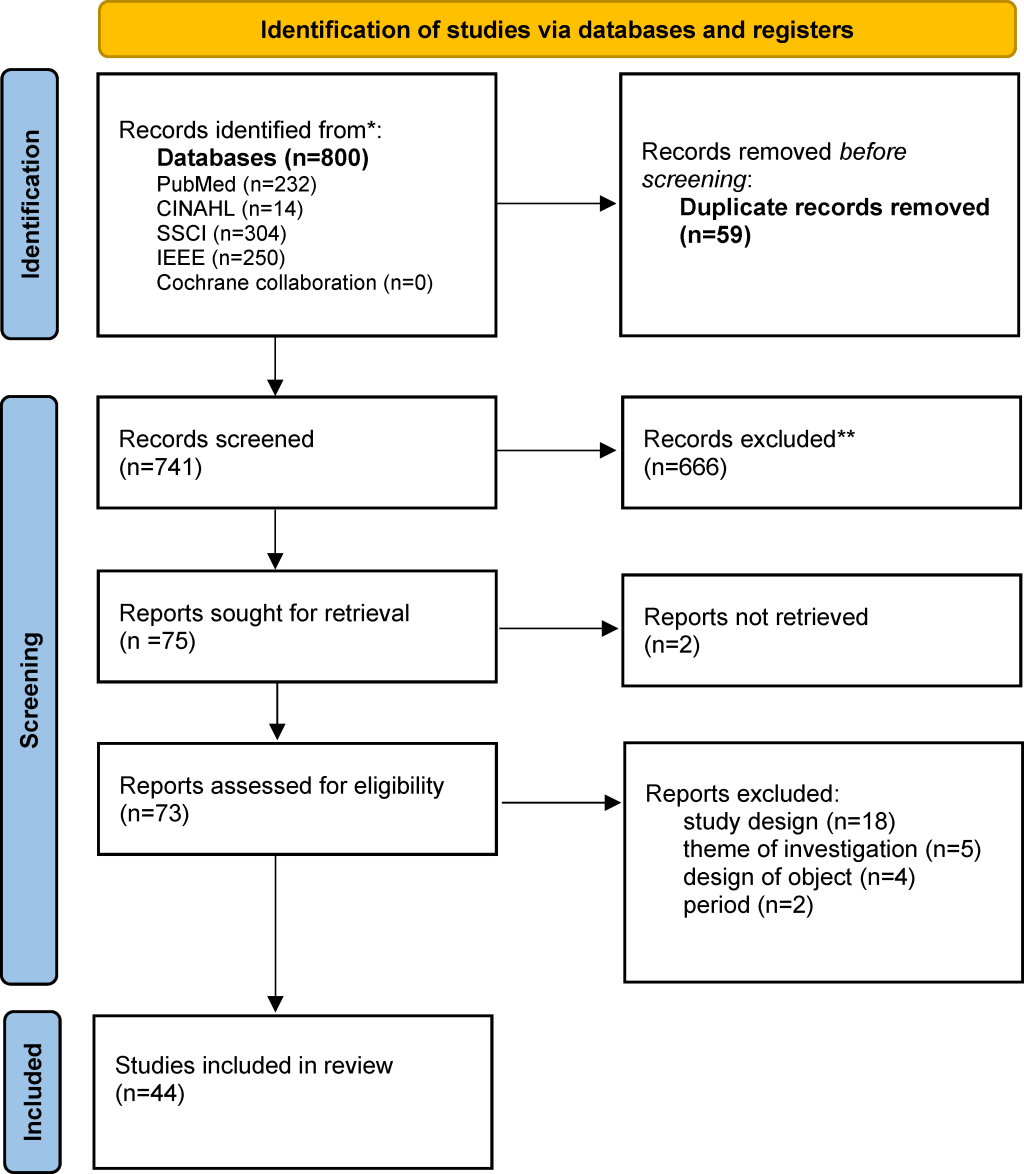
PRISMA (Preferred Reporting Items for Systematic Reviews and Meta-Analyses) flowchart for scoping reviews. *Number of records identified from each database. **Number of records excluded.

An initial pool of 800 articles was identified for screening (duplicates removed n=59, 7.4%). After title and abstract screening, 666 (83.3%) articles were excluded based on the predefined criteria related to theme and publication period. A total of 75 (9.4%) records were sought for retrieval. Although the original authors were contacted, 2 (0.3%) studies could not be included due to missing full texts. The remaining 73 (9.1%) articles underwent full-text review, leading to the exclusion of 29 (3.6%) studies due to study design, theme of investigation, and design of object or period. Ultimately, 44 (5.5%) articles met the inclusion criteria. The entire selection process is summarized in the PRISMA (Preferred Reporting Items for Systematic Reviews and Meta-Analyses) flowchart [15] (Figure 1).

### Description of Included Studies

**Figure 2.**
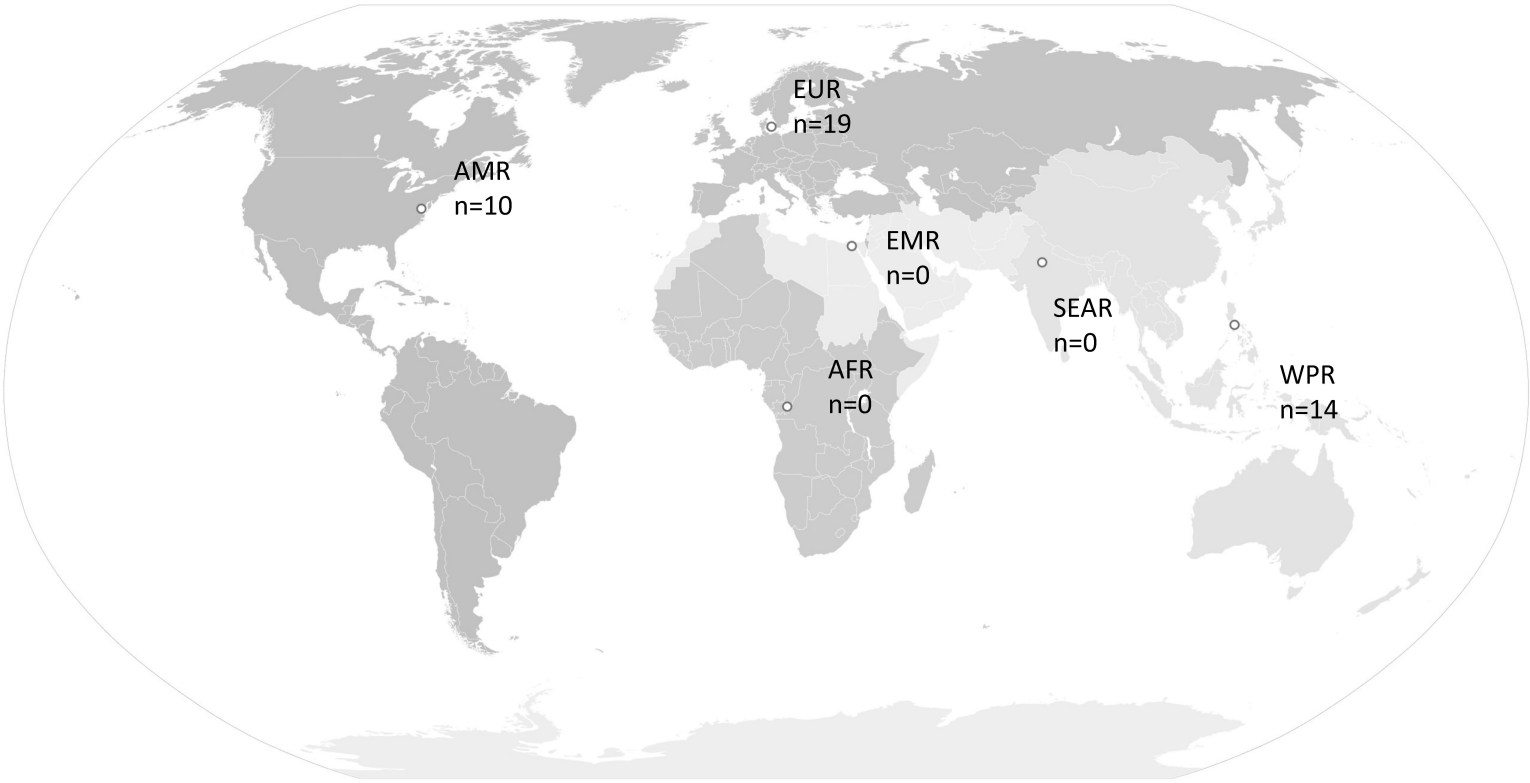
Geographical distribution of included studies. Countries were grouped according to the World Health Organization regions. AFR: African Region; AMR: Region of the Americas; EMR: Eastern Mediterranean Region; EUR: European Region; SEAR: South-East Asia Region; WPR: Western Pacific Region.

Of the 800 articles screened, 44 (5.5%) met the predefined inclusion criteria. Geographically, the research was predominantly conducted in Europe (n=19, 43.2%; Figure 2).

Most of these studies were research reports (n=36, 81.8%). Sample sizes varied considerably across the included studies, ranging from a minimum of 1 participant to a maximum of 43 participants. A total of 5 (11.4%) studies did not report the number of participants. Most evaluations of smart walkers were conducted exclusively or partially in asymptomatic populations (n=29, 65.9%), with half (n=22, 50%) of the studies involving younger adults. A total of 14 (31.8%) studies involved older adults (6 studies without specifying age group and 1 without any person; Table 1).

**Table 1.** Overview of study characteristics grouped by year, setting, study type, and population.

Study, country	Year of publication	N	Setting	Study type	Population
Alves et al [16], Portugal	2016	6	Clinical	Research report	Symptomatic and younger adults
Caetano et al [17], Portugal	2016	Not specified	Clinical and rehabilitation	Research report	Not specified
Ballesteros et al [18], Netherlands, Spain, and Italy	2016	43	Laboratory and clinical	Research report	Symptomatic, asymptomatic, younger, and older adults
Hellström et al [19], Sweden	2016	12	Laboratory	Research report	Asymptomatic, symptomatic, and older adults
Valadão et al [13], Brazil	2016	Not specified	Laboratory	Research report	Asymptomatic^a^
Ballesteros et al [20], Spain	2017	19	Rehabilitation	Research report	Symptomatic^a^
Mun et al [21], Singapore	2017	10	Laboratory	Research report	Asymptomatic and younger adults
Ohnuma et al [22], Japan	2017	5	Not specified	Research report	Asymptomatic and older adults
Paulo et al [23], Portugal	2017	30	Laboratory	Research report	Asymptomatic and younger adults
Taghvaei et al [24], Japan	2017	4	Not specified	Research report	Asymptomatic and younger adults
Zhang and Ye [25], not applicable	2017	9	Laboratory	Research report	Not specified
Werner et al [26], Germany	2018	42	Clinical	Randomized controlled trial	Asymptomatic, symptomatic, and older adults
Andreetto et al [27], Italy	2019	29	Laboratory	Research report	Asymptomatic, symptomatic, younger, and older adults
Ferrari et al [28], Italy	2019	29	Laboratory	Research report	Asymptomatic, symptomatic, and older adults
Li et al [29], Japan	2019	3	Laboratory	Research report	Asymptomatic and younger adults
Moreira et al [30], Portugal	2019	3	Rehabilitation	Research report	Symptomatic^a^
Pérez-Rodríguez et al [31], Spain	2019	34	Clinical	Randomized controlled trial	Symptomatic and older adults
Sato et al [32], Japan	2019	11	Community dwelling	Research report	Asymptomatic and older adults
Scheidegger et al [33], Columbia	2019	5	Laboratory	Research report	Symptomatic and younger adults
Sierra et al [10], Columbia	2019	7	Laboratory	Research report	Asymptomatic and younger adults
Werner et al [34], Germany	2019	25	Laboratory	Research report	Symptomatic and older adults
Alazem et al [35], Canada	2020	5	Laboratory	Research report	Symptomatic, children, and younger adults
Jiménez et al [36], Brazil	2020	15	Laboratory	Research report	Not specified
Yeoh et al [37], Japan	2020	18	Not specified	Research	Asymptomatic and younger adults
Werner et al [38], Germany	2020	33	Laboratory	Research report	Asymptomatic and older adults
Zhao et al [39], China	2020	Not specified	Laboratory	Research report	Not specified
Chang et al [40], Taiwan	2021	Not specified	Laboratory	Research	Asymptomatic^a^
Mostofa et al [41], United States	2021	11	Community dwelling	Research report	Asymptomatic and older adults
Moustris et al [42], Greece	2021	32	Rehabilitation	CCT^b^	Asymptomatic, symptomatic, younger, and older adults
Orenius et al [43], Finland	2021	19	Not specified	CCT	Older adults^c^
Sierra et al [44], Columbia	2021	10	Laboratory	Research report	Asymptomatic and younger adults
Sierra et al [45], Columbia	2021	10	Laboratory	Research report	Symptomatic and older adults
Zhang et al [46], China	2021	8	Laboratory	Research	Asymptomatic and younger adults
Fernandez-Carmona et al [47], Spain	2022	11	Laboratory	Observational study	Younger and older adults^c^
Palermo et al [48], Portugal	2022	14	Laboratory	Research report	Asymptomatic and younger adults
Jacobs et al [49], United States	2023	11	Laboratory	Observational study	Asymptomatic and younger adults
Schwarz et al [50], Germany	2023	Not applicable	Laboratory	Research report	Younger adults^c^
Wang et al [51], China	2023	1	Laboratory	Research report	Asymptomatic^a^
Gong et al [52], China	2024	3	Laboratory	Research	Asymptomatic and younger adults
Jiang et al [53], China	2024	3	Laboratory	Observational study	Asymptomatic and younger adults
Machado et al [54], Brazil	2024	20	Laboratory	Observational study	Asymptomatic and younger adults
Mori et al [55], Japan	2024	5	Laboratory	Research report	Asymptomatic and younger adults
Sierra et al [56], United Kingdom	2024	14	Laboratory	Research report	Asymptomatic and younger adults
Zhang et al [57], Japan	2024	20	Community dwelling	Research report	Asymptomatic^a^

a No information about age.

b CCT: controlled clinical trial.

c No information about health status.

### Settings and Participants

The studies were conducted across various settings. Investigations involving smart walkers were most frequently conducted in laboratory-based environments (n=30, 68.2%).

**Figure 3.**
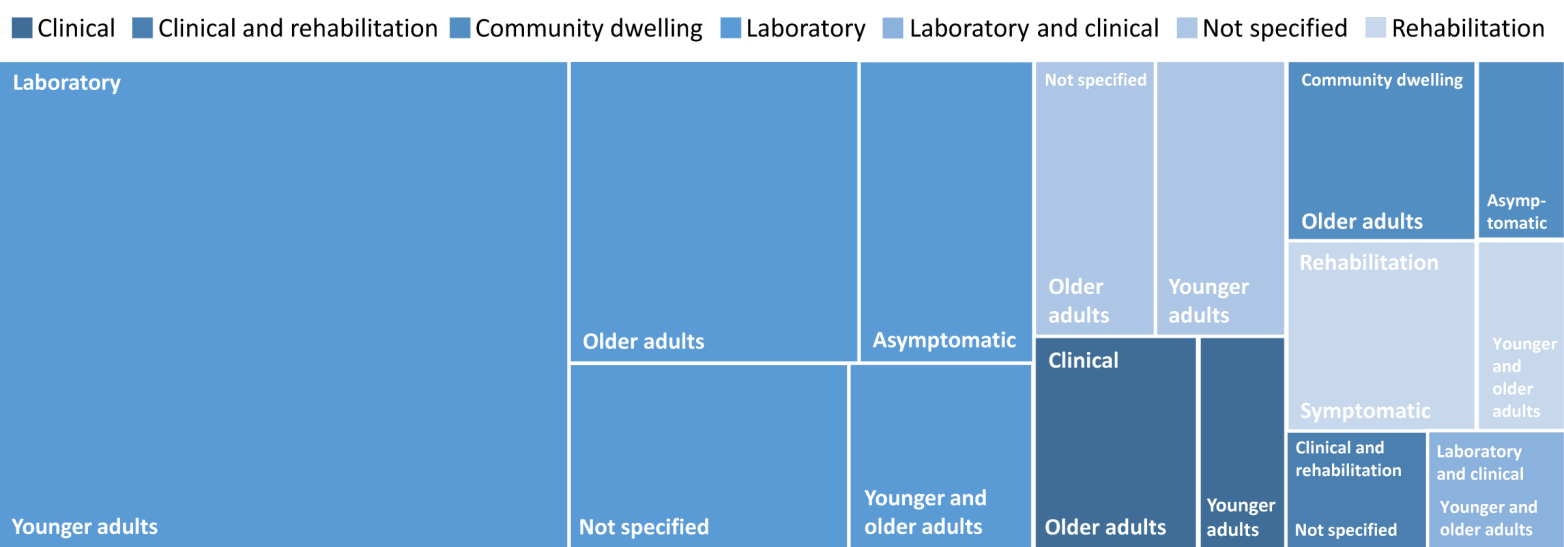
Proportion of different settings and participants.

Four groups of participants were evident in the articles included: symptomatic children or younger adults (n=5, 11.4%), asymptomatic younger adults (n=19, 43.2%), symptomatic older adults (n=8, 18.2%), and asymptomatic older adults (n=9, 20.4%). Several articles did not specify the age (n=6, 13.6%) or health status (n=5, 11.4%) of participants. A total of 4 (9.1%) articles provided no information on the age and health status. As there were studies with multiple participant groups, the number of participant groups exceeded the number of articles. The group of participants consisting of asymptomatic younger adults was the most frequently studied group across the articles (n=19, 43.2%; Table 1, Figure 3).

### Walker-Specific Sensor Technologies and Functionalities

The included articles described various types of sensors and functions that constitute the extent of smart technology integrated into modern walkers. The sensors used were fundamental to the operation of these intelligent walkers. They encompassed a range of sensor types, including camera systems (2D cameras, depth cameras, and infrared cameras), optical sensors that used laser lights (2D laser scanners and light detection and ranging [LiDAR]), force and load sensors, ultrasound sensors, and inertial measurement units (IMUs; Table 2).

Among the sensor modalities reported, camera- and LiDAR-based sensors were the most prevalent, accounting for half of the implementations. Camera sensors were described either as standard 2D cameras (n=11, 25%) [10,17,23,24,27,28,31,33,42,44,56] or depth cameras. Depth cameras were used for multiple purposes, including gait analysis (n=3, 6.8%) [16,41,55] and intention recognition (n=3, 6.8%) [23,42,52], by capturing gaze direction. Additionally, depth cameras were used for collision avoidance through object detection (n=4, 9.1%) [17,23,25,42], and 1 (2.3%) study specifically described the use of depth cameras for fall prediction purposes [24]. LiDAR-based sensors can be categorized according to their primary functions: gait analysis (n=11, 25%), collision detection (n=9, 20.5%), and navigation (n=5, 11.4%).

In addition, load sensors (n=10, 22.7%) and ultrasonic sensors (n=11, 25%) were among the most frequently cited sensor modalities in the literature. Furthermore, load and force sensors were typically integrated into the handlebars of smart walkers (n=10, 22.7%) [10,17,18,23,39,44,45,47,48,56]. These sensors were described as “the simplest approach to onboard sensor-based gait analysis” [47] and were used to assess the user’s balance during ambulation [17]. Zhao et al [39] further detailed handgrip pressure sensors that can potentially prevent falls by detecting abnormal pressure or rapid pressure changes, indicating a fall risk. For instance, the smart walker developed by Ballesteros et al [18] incorporated force sensors in the handlebars alongside encoders to measure wheel rotation.

Beyond handlebar-mounted sensors, force sensors were also embedded elsewhere in the walker’s structure, such as in the frame, forearm supports, or chest pads (n=7, 15.9%) [16,32,46,51-54]. Sensors placed within the frame measured the user’s weight load, providing critical data for calculating body weight support (n=3, 6.8%) [46,51,52]. Additionally, Machado et al [54] and Jiménez et al [36] reported on force sensors located in forearm supports, which were used to infer the user’s intention to move, thereby enabling more responsive and adaptive assistance.

Ultrasound sensors were described in a quarter of the articles (n=11, 25%) and were used for collision avoidance [10,13,27,28,30,31,33,41,44,45,48]. IMUs were described with a similar frequency (n=12, 27.3%) [10,26,27,30,33,36,39,44,45,54-56]. An analysis of their functions revealed that they were mainly used to determine position and orientation, measure accelerations, record angular velocities, or support odometry sensors.

**Table 2.** Sensors and their function in smart walkers.

Functions	Sensors
Safety and collision detection	LiDAR^a^, microphone, ultrasound sensors, camera, and infrared
Navigation	LiDAR, optical encoder, and IMUs^b^
Propulsive support and reduction of speed	Odometry sensors, force and load sensors, and IMUs
Body weight support	Force and load sensors
Intention to move	Force and load sensors and camera (detecting gaze)
Movement analysis (gait, balance, and postural control)	LiDAR, depth camera, and 2D laser range finder
Fall risk detection and falls	3D load sensors and depth camera

a LiDAR: light detection and ranging.

b IMU: inertial measurement unit.

With regard to sensory-based feedback generated, the following distinctions can be reported: user feedback, which provides relevant information on, for example, navigation details or collision warning during the use of the smart walker (Table 3), or observer feedback, which, for example, provides valuable additional information such as gait-related or system-relevant measurement data (Table 4) to the observer or therapist.

**Table 3.** User feedback mechanisms (multiple entries possible; N=44).

Category of observer feedback	Entries, n (%)
Haptic or tactile	10 (22.7)
Visual, user interface or navigation	13 (29.6)
Assistive control-based feedback	7 (15.9)
Virtual, multimodal or social	5 (11.4)
No user feedback	10 (22.7)
Unclear	3 (6.8)

**Table 4.** Observer feedback mechanisms (multiple entries possible; N=44).

Category of observer feedback	Entries, n (%)
Objective measurement data, gait analysis and sensor data	27 (61.4)
User experience, interaction and system data	14 (31.8)
No observer feedback	10 (22.7)
Unclear	4 (9.1)

## Discussion

### Principal Findings

This scoping review was conducted to provide a comprehensive overview of the current application settings, sensors, and functionalities of smart walkers.

Various sensors, such as LiDAR, depth camera, and 2D laser range finder, which could be used for a clinical assessment of the gait pattern or for measuring balance, have been integrated into smart walkers. Analysis of the intended use of sensors revealed a wide variety of applications. For instance, IMUs were mainly used to determine position and orientation, measure system accelerations, record angular velocities, and support odometry. The gait analysis functionality and the validity of the measured parameters depend on the sensor types used and their placement on the smart walker.

Analysis of the included studies indicates that validation data for gait-related sensor technology are often missing or only partially reported. Only 8 (22.9%) of the 35 studies measuring gait-relevant data used reference systems such as the one used in the study by Werner et al [34], IMU-based systems (MTw Awinda, Xsens Technologies, B.V.) [48], or a marker-based camera system operating at 120 Hz (Vicon motion analysis system) [44]. These studies provided the most methodologically robust evidence regarding measurement accuracy and are best suited for comparison with established clinical measurement systems. An additional 17 (38.6%) studies reported some form of validation but without a reference standard, mostly in the form of internal error measures (eg, root mean square error, difference metrics, and trial comparisons). While these data provide valuable technical insights, they allow only limited conclusions regarding external validity. Some articles provided no information on the validation of gait sensors, which is partly due to the studies’ focus. In many studies, the primary objective was different, such as navigation, safety, human-robot interaction, or technology design, meaning that gait analysis was only of marginal or no relevance. Overall, there is a clear gap between technical innovation and methodological transparency: although smart walker systems increasingly integrate complex sensor sets, validation against established gold standards remains uncommon. This lack of consistent validation limits the transferability of findings to clinical practice.

This review demonstrates that smart walkers that have been investigated in laboratory-oriented settings and among user populations are not representative of real-world applications. Most research has taken place in controlled laboratory environments and primarily involves asymptomatic and/or younger users of smart walkers. In contrast, older adults (representing an increasing global aging population) are key drivers of the expanding market for walking aids and walkers [58]. It is undisputed that younger adults have fewer functional limitations, better balance abilities, and overall exhibit fewer fall-related risk factors than older adults. As noted by Maranesi et al [59], research in this field should move beyond focusing solely on the technical functionality of gait devices and walkers. Our results illustrate a gap in effectively incorporating these devices into relevant settings, such as clinical environments or the daily lives of end users. This underscores the need for a comprehensive understanding of the physical, social, and cognitive needs of older adults. In 2025, a funded research and development initiative was launched in Germany to develop a prototype of a smart walker. The SmartRoll project adopts a user-centered study design, systematically identifying the needs of patients, professional caregivers, and physiotherapists [60]. These insights are subsequently evaluated with the target population—patients on an acute geriatric ward—as part of an integrated research and development effort. Additionally, the project involves the integration and testing of sensors capable of performing gait analysis in this vulnerable group of older adults. This scoping review indicates that the search for smart walkers in the clinical setting implies their use for research purposes within clinical environments. The relevance of smart walkers in clinical contexts is driven by the urgent need to address age-related skill decline [26] and mobility impairment [45]. This paper is embedded in a study that examines the current relevance of deploying smart rollators in clinical settings.

Older individuals who rely on assistive devices often do so due to underlying health conditions or following adverse events such as falls. Consequently, it is not surprising that users of walkers exhibit a fourfold increased risk of falls compared to individuals walking without such aids [61]. This population is particularly vulnerable during acute hospital stays. The use of smart walkers equipped with gait analysis and balance measurement functions could potentially be used in hospital settings to assess fall risk among patients. Indicators of fall risk are primarily identified through parameters that suggest an unstable gait balance and gait pattern. These parameters include, but are not limited to, foot angle at contact, step length, maximum foot height, and a high SD in step characteristics [1].

It should be noted that research comparing 2-, 3-, and 4-wheeled walkers is limited. Two-wheeled walkers often need to be partially lifted, requiring strength and coordination and affecting gait parameters. As the poor maneuverability and lower lateral stability of 2-wheeled walkers may pose additional risk factors [62], they were excluded from this review.

### Conclusions

The smart walkers described in the literature were predominantly tested in asymptomatic and young populations. Developing smart walkers that explicitly address the specific needs of older adults—through systematic requirements analyses—and iterative testing throughout the development process remains an ongoing challenge. Integrating these steps as core components of research and development projects is essential to enhance clinical relevance.

## Supplementary material

10.2196/78480 Multimedia Appendix 1 Search strings for the SSCI, CINAHL, IEEE Xplore, and Cochrane Library databases.

10.2196/78480 Checklist 1 PRISMA-ScR checklist.

## Abbreviations

IMU: inertial measurement unit
LiDAR: light detection and ranging
PRISMA: Preferred Reporting Items for Systematic Reviews and Meta-Analyses
PRISMA-ScR: Preferred Reporting Items for Systematic Reviews and Meta-Analyses extension for Scoping Reviews
