# Supplementary material for: Functions and Sensors of Smart Walkers From 2015 to 2024: Scoping Review
Source: JMIR Rehabil Assist Technol. 2026 May 26;13:e78480. doi: 10.2196/78480 (PMC13207166; doi:10.2196/78480)
Supplement: Multimedia Appendix 1 [file rehab-v13-e78480-s001.docx]

Search string in Databases SSCI, CINAHL, IEEE, Cochrane Library

| **SSCI** | |
| --- | --- |
| 1 walker* (Title)  2 rollator* (Title)  3 device supported (Title)  4 #1 OR # 2 OR # 3  5 smart (Title)  6 intelligent (Title)  7 robot* (Title)  8 artificial (Title)  9 ai (Title)  10 #5 OR #6 OR #7 OR #8 OR #9 | 11 DNA OR  12 device-based OR  13 molecular OR  14 Covid* OR  15 exoskeleton OR  16 teaching OR  17 walker* and avant* (Title)  18 #11 OR #12 OR #13 OR #14 OR #15 OR #16 OR #17  19 #4 AND #10 NOT #18 |
| **CINAHL** | |
| AB (walker AND smart NOT exoskeleton) OR (walker AND ai NOT exoskeleton) OR (walker AND artificial NOT exoskeleton) OR (walker AND intelligent NOT exoskeleton)  + AB (walker AND Robot* NOT exoskeleton)  + AB (rollator AND smart NOT exoskeleton) OR (rollator AND ai NOT exoskeleton) OR (rollator AND artificial NOT exoskeleton) OR (rollator AND intelligent NOT exoskeleton)  + AB (rollator AND Robot* NOT exoskeleton) | |
| **IEEE** | |
| ‘Smart Walker’; Filter: 2015-2024; Journals; | |
| **Cochrane Library** | |
| ‘smart’ OR ‘intelligent’ OR ‘robot*’ OR ‘artificial’ OR ‘ai’ in Title Abstract Keyword AND ‘walker*’ OR ‘rollator*’ OR ‘device supported’ in Title Abstract NOT ‘DNA’ OR ‘device-based’ OR ‘molecular’ OR ‘Covid*’ OR ‘exoskeleton’ OR ‘teaching’ OR ‘walker* and avant’ Title Abstract Keyword | |
